# Omega-3 Fatty Acids in Modern Parenteral Nutrition: A Review of the Current Evidence

**DOI:** 10.3390/jcm5030034

**Published:** 2016-03-07

**Authors:** Stanislaw Klek

**Affiliations:** Stanley Dudrick’s Memorial Hospital, General Surgery Unit, Skawina 32-050, Poland; klek@poczta.onet.pl; Tel.: +48-12-424-80-07

**Keywords:** parenteral nutrition, omega-3 fatty acids, omega-3 PUFA, lipid emulsions

## Abstract

Intravenous lipid emulsions are an essential component of parenteral nutrition regimens. Originally employed as an efficient non-glucose energy source to reduce the adverse effects of high glucose intake and provide essential fatty acids, lipid emulsions have assumed a larger therapeutic role due to research demonstrating the effects of omega-3 and omega-6 polyunsaturated fatty acids (PUFA) on key metabolic functions, including inflammatory and immune response, coagulation, and cell signaling. Indeed, emerging evidence suggests that the effects of omega-3 PUFA on inflammation and immune response result in meaningful therapeutic benefits in surgical, cancer, and critically ill patients as well as patients requiring long-term parenteral nutrition. The present review provides an overview of the mechanisms of action through which omega-3 and omega-6 PUFA modulate the immune-inflammatory response and summarizes the current body of evidence regarding the clinical and pharmacoeconomic benefits of intravenous *n*-3 fatty acid-containing lipid emulsions in patients requiring parenteral nutrition.

## 1. Introduction

Intravenous (IV) lipid emulsions (LE) are an integral component of parenteral nutrition (PN) regimens. The earliest LE, subsequently referred to as “first generation” LE, were derived from soybean or cottonseed oil. Soybean oil is rich in omega-6 (*n*-6) polyunsaturated fatty acids (PUFA) and provides high amounts of linoleic acid (LA) and moderate amounts of α-linolenic acid (ALA), with an *n*-6/*n*-3 ratio of approximately 7:1 [1,2,3,4,5]. These early emulsions, which were primarily used as an efficient non-glucose energy source to reduce the adverse effects of high dextrose intake, have two main functions: to provide a source of energy and supply essential fatty acids [6].

While the cottonseed oil-based LE (Lipomul, USA) has been permanently removed from the market [6], the soybean oil-based LE Intralipid^®^ (Fresenius Kabi, Germany) has been used worldwide since its introduction in 1962 and has been proven to be a safe and well tolerated emulsion [3,7,8,9,10]. However, a potential disadvantage with existing soybean oil emulsions is their relatively high content of *n*-6 PUFA (polyunsaturated fatty acids), in particular LA [2,11,12]. The emergence of evidence suggesting that *n*-6 polyunsaturated fatty acids might be pro-inflammatory and immunosuppressive led to the development of more complex lipid emulsions consisting of a mixture of different oils [13]. As a result, the latest generation of *n*-3 fatty acid-containing LE provide a more balanced combination of *n*-6 and *n*-3 fatty acids, with an *n*-6/*n*-3 ratio in the range of 2:1–4:1 [14,15]. For this reason, second and third generation IVLE contain not only soybean oil but alternative lipid sources such as medium-chain triglycerides (MCT), olive oil and/or fish oil/*n*-3 fatty acids [2,5]. In particular, *n*-3 fatty acid-containing IVLE have received considerable attention due to their ability to modulate key metabolic functions, including inflammatory response, coagulation, and cell signaling [5].

The present review provides a brief overview of the mechanisms of action and the role of *n*-6 and *n*-3 PUFA in modulating the immune-inflammatory response. The subsequent sections summarize the health benefits associated with *n*-3 long-chain (LC)-PUFA as well as the available evidence regarding the clinical and economic advantages of PN with *n*-3 fatty acids.

## 2. Classification and Mechanisms of Action

Fatty acids are classified according to their structure, carbon chain length (short, medium, or long), degree of saturation (number of double bonds), and the location of double bonds (counted from the methyl carbon of the hydrocarbon chain) [2,3,4,5,6]. As a common structural feature of all *n*-3 PUFAs, the double bond closest to the methyl terminus of the acyl chain of the fatty acid is located on carbon 3 [16]. The parent fatty acid of the *n*-3 family is ALA. The active ingredients in fish oil are the *n*-3 LC-PUFAs, eicosapentaenoic acid (EPA, C20:5 *n*-3) and docosahexaenoic acid (DHA, C22:6 *n*-3), as well as the *n*-6 LC-PUFA arachidonic acid (AA, C20:4 *n*-6). All are involved in the generation of pro- and anti-inflammatory lipid mediators. While AA exerts a pro-inflammatory effect, EPA and DHA have the ability to reduce inflammation through anti-inflammatory and immunomodulatory mechanisms [13,17,18,19].

The oil obtained from the flesh of oily fish or livers of lean fish is commonly referred to as “fish oil” and it has the distinctive characteristic of being rich in *n*-3 LC-PUFAs [1]. The effects of emulsions described as “fish oil rich” or “*n*-3-rich/containing” are attributable to the LC-PUFAs EPA, DHA, and in some cases ALA. Various oily fish contain different amounts of *n*-3 fatty acids, as do the various fish oils. This also applies to the content of EPA and DHA in enteral and parenteral products; hence, it is important to analyze the ingredients of PN admixtures to ensure they provide the appropriate amount of EPA and DHA.

In Europe there are currently three available IVLE products containing *n*-3 LC-PUFAs: Omegaven^®^ (Fresenius Kabi, Germany), Lipoplus^®^/Lipidem^®^ (B. Braun, Germany), and SMOFlipid^®^ (Fresenius Kabi, Germany). Omegaven^®^ is a 10% fish oil emulsion supplement. Lipoplus^®^ contains a mix of 50% MCT, 40% soybean oil, and 10% fish oil, and SMOFlipid^®^ is a 4-oil mixture of 30% soybean oil, 30% MCT, 25% olive oil, and 15% fish oil.

## 3. Fatty Acids: An Overview of Clinical Impact

Fatty acids represent not only a significant energy source and a human body energy store, but they are also necessary for proper biologic function. Fatty acids are key structural components of cell membranes (phospholipids), assuring membrane integrity and fluidity. They also serve as precursors of bioactive mediators such as eicosanoids (prostaglandins, leukotrienes, and thromboxanes) and steroid hormones (cholesterol) [1]. Finally, lipids regulate the expression of a variety of genes and modulate cell signaling pathways (apoptosis, inflammation, and cell-mediated immune responses). Therefore, lipids can modulate metabolic processes at local, regional, and distant sites [1].

### 3.1. Fatty Acids and Immune-Inflammatory Response

Inflammation is part of the complex physiological response to harmful stimuli such as pathogens, damaged cells, toxins, and irritants [19]. The primary functions of inflammation are to eliminate the initial cause of injury, launch defense mechanisms, remove necrotic cells and tissues, and initiate tissue repair [1,19]. Properly regulated inflammation represents an efficient physiological mechanism that protects the host from infection and other insults and is thus essential to health. Conversely, excessive pathological inflammation may cause irreparable damage to host tissues and ultimately lead to sepsis—the complex systemic inflammatory host response to an infection [20].

Inflammation can be classified as either acute or chronic according to the duration of the response. It can also be categorized according to the intensity of the process. In 1992, the American College of Chest Physicians and the Society of Critical Care Medicine introduced definitions for systemic inflammatory response syndrome (SIRS), sepsis, severe sepsis, septic shock, and multiple organ dysfunction syndrome [21]. SIRS is nonspecific and can be caused by ischemia, inflammation, trauma, infection, or multiple combined insults. It is defined as two or more of the following: temperature >38 °C (100.4 °F) or <36 °C (96.8 °F), heart rate >90 beats per minute and/or respiratory rate >20 breaths per minute or arterial carbon dioxide tension (PaCO2) <32 mm Hg [21]. It is important to recognize that SIRS is not always related to infection; accordingly, it cannot be treated the same way. Sepsis, by contrast, always stems from infection; indeed, it is defined as the presence of harmful bacteria and bacterial toxins in blood and tissues (sepsis = SIRS + infection). Severe sepsis is a condition in which there is organ dysfunction, hypotension, or hypoperfusion. If it progresses, septic shock may develop, potentially leading to multiple organ dysfunction syndrome (formerly known as multiple organ failure) and death.

During critical illness, increased production of reactive oxygen species and inflammatory mediators occurs along with a reduction in antioxidant activity, which is partly due to preexisting nutritional deficiencies and/or suboptimal provision of clinical nutrition [6]. This state of imbalance can cause tissue damage and may play an important role in the development of sepsis and multiple organ failure [6]. Therefore, the primary goals of treatment in critically ill patients should be to decrease the pro-inflammatory response during the catabolic phase of illness and enhance immune defense mechanisms. PN should always be considered as part of any such intervention if enteral intervention is impossible or insufficient [22]. The *n*-3 fatty acids EPA and DHA exhibit strong anti-hyperinflammatory and immunomodulatory effects via direct and indirect mechanisms and may thus be of benefit in patients at risk of hyperinflammation and sepsis [2,13,16].

The incorporation of PUFA into the phospholipids of cell membranes ensures the maintenance of membrane fluidity and the adequate function of membrane proteins. Through the formation of membrane rafts, *n*-3 PUFA are also involved in the inhibition of tumor growth [23,24]. The ratio of *n*-6/*n*-3 PUFA released from the hydrolysis of membrane phospholipids influences the synthesis of eicosanoid mediators such as prostaglandins (PGs), thromboxanes (TXs), and leukotrienes (LTs). The *n*-6 PUFA AA gives rise to 2-series PGs, TXs, 5-hydroxy-eicosatetraenoic acid (HETE), and 4-series LTs, thereby contributing to the inflammatory process and suppressing cell-mediated immunity [25,26,27,28]. Conversely, enzymatic conversion of the *n*-3 fatty acid EPA results in the production of 3-series PGs and TXs and 5-series LTs, all of which are less potent than the AA-derived mediators [16,24,28].

The recent discovery of families of novel pro-resolving lipid mediators such as resolvins, protectins, maresins, and lipoxins has shed new light on the role of *n*-3 PUFAs in the inflammatory process [29,30,31,32]. Pro-resolving lipid mediators derived from EPA are designated resolvins of the E series (RvE), while those derived from DHA are designated resolvins of the D series (RvD) [33]. The latter include the neuroprotectins/protectins (NPD1/PD1) and the maresins (MaR). These mediators have been shown to limit neutrophilic infiltration and enhance macrophage resolution responses, thus playing a role in diseases characterized by excessive uncontrolled inflammation [34,35,36,37,38,39,40,41]. Notably, their generation is linked to the fatty acid composition of cellular membranes and can therefore be effectively increased with *n*-3 fatty acid supplementation [37].

Exposure of inflammatory cells to *n*-3 PUFA *in vitro* attenuates chemotaxis of human neutrophils and monocytes and decreases expression of certain adhesion molecules on the surface of monocytes, macrophages, lymphocytes, and endothelial cells [20]. By coupling with surface or intracellular “fatty acid receptors”, the LC-PUFA or their oxidized derivatives can regulate gene expression via the activation of transcription factors such as peroxisome proliferator-activated receptor (PPAR)-γ and nuclear factor-kappa B (NF-κB). *N*-3 LC-PUFA also modulate the inflammatory processes by inhibiting the production of pro-inflammatory cytokines and other pro-inflammatory proteins induced via activation of NF-κB in response to exogenous inflammatory stimuli. It appears that this effect is at least partly mediated by G-protein coupled cell surface receptors [20,24]. Consequently, the use of EPA and DHA leads to a more balanced immune response, which may result in faster resolution of inflammation [4,13,17,18].

## 4. Physiological Effects and Associated Health Benefits of *n*-3 LC-PUFA

### 4.1. Cardioprotective, Antihypertensive, and Antithrombotic Effects

In animal models of atherogenesis, *n*-3 fatty acids inhibit the hepatic synthesis of triglycerides [1]. EPA and DHA partly replace AA in membrane phospholipids; therefore, *n*-3 fatty acids may improve membrane structure, ligand/receptor binding, enzyme secretion, antigen presentation, and activation of intracellular signaling pathways [1]. By reducing the availability of AA-derived fatty acids as a substrate for eicosanoid synthesis by cyclooxygenase and lipoxygenase in platelets, monocytes, and macrophages, EPA and DHA delay platelet aggregation and progression of atherogenesis [10].

Consuming *n*-3 PUFA from fish oil is beneficial in the prevention of cardiovascular disease (CVD) and associated complications, as demonstrated by randomized control trials (RCTs) investigating secondary prevention in heart disease patients as well as epidemiological studies in populations that traditionally consume large quantities of sea fish as a part of their daily diet (e.g., Inuit) [42,43]. Potential underlying mechanisms include the inhibition of thrombogenesis and cytokine-dependent inflammation [42]. At total doses >3 g/day, EPA + DHA reduces CVD risk factors by decreasing plasma triglycerides, blood pressure, platelet aggregation, and inflammation and improving vascular reactivity [43].

### 4.2. Anticancer and Anti-Cachectic Effects and Inhibition of Tumor Growth

*N*-3 PUFA have been shown to attenuate cell growth and induce apoptosis in a variety of human cancer cell lines such as colonic, pancreatic, prostate, and breast cancer [44]. Through their effects on eicosanoid metabolism, *n*-3 PUFA may also decrease “sprouting angiogenesis”, suppress endothelial cell proliferation, decrease tumor micro-vessel density, and even decrease tumor growth [45,46,47,48,49]. Research indicates that *n*-3 fatty acids act synergistically with chemotherapeutic agents and may also be used to enhance tumor radiosensitivity [50,51,52]. In patients with cancer-related cachexia, the pro-inflammatory state can be modulated by suppressing the inflammatory milieu and the release of pro-inflammatory mediators such as cytokines and prostaglandins by means of fish oil-based preparations, thus allowing nutrition to have an anabolic effect [53]. The underlying mechanisms for these effects include the incorporation of *n*-3 fatty acids into biological membranes as well as the modulation of the expression of proteins involved in the regulation of cell cycle and apoptosis, such as Bcl-2, Bax, and c-Myc [23,45,54]. More studies are needed, however, to fully assess the effects of *n*-3 PUFA in cancer treatment and prevention.

### 4.3. Visual and Cognitive Development

*N*-3 PUFA may reduce the risk of neurological disorders by influencing neuronal membranes and the activities of membrane-bound enzymes, receptors, and transporters [55]. Moreover, EPA and DHA can affect neurotransmission, including dopaminergic, noradrenergic, serotoninergic, and GABAergic neurotransmission in specific brain regions [56,57]. DHA, together with AA, is crucial for the development and maintenance of normal structure and function of the central nervous system (CNS). During fetal development DHA is taken up via the placenta and accumulates in the brain where it is required for the proper function of cholinergic neurotransmission [57,58,59,60]. Additionally, DHA may protect the brain from free radical and reactive oxygen species by enhancing the activity of cerebral catalase and glutathione peroxidase [61]. DHA may also be important for the efficient regeneration of axons and dendrites following neuronal injury [55].

The putative effects of *n*-3 PUFA on CNS function are mediated not only through an effect on the physicochemical properties of neural membranes but also through activation of transcription factors such as PPAR-γ [62]. AA, EPA, and DHA play a major role in protecting neuronal cells in the brain via inhibition of tumor necrosis factor (TNF)-α synthesis, thereby augmenting acetylcholine and endothelial nitric oxide (NO) formation and enhancing glucose uptake by neuronal cells, resulting in improved memory [53]. Epidemiological data suggest that a low dietary intake of *n*-3 LC-PUFA is a risk factor for Alzheimer disease [63]. Notably, studies have shown that the intake of fish oil lowers the risk of dementia by reducing the synthesis of proinflammatory cytokines and inhibiting the activities of phospholipase A2 and caspase A1 [64,65].

The effects of EPA and ethyl-EPA intake in schizophrenic patients have been investigated in a number of RCTs [63]. Overall, the results suggest a potential benefit; however, due to the limited size and duration of the studies it remains unknown whether the benefit is clinically meaningful. Research also suggests that *n*-3 LC-PUFA may confer a benefit in certain cases of depression; however, the routine use of *n*-3 LC-PUFA for the treatment of major depression cannot yet be recommended. Further research is necessary to establish the efficacy, appropriate dosing, and active components (EPA, DHA, or both) of *n*-3 LC-PUFA in patients with major depression [55].

### 4.4. Lipid Metabolism and Insulin Sensitivity

Fish oil supplementation has been shown to produce a clinically significant, dose-dependent reduction in fasting blood triglycerides and normalize serum lipid concentrations, including high density lipoproteins (HDL) and low density lipoproteins (LDL), in patients with hyperlipidemia [66]. Fish oil may also be beneficial in individuals with diabetes mellitus. In patients with type 2 diabetes, fish oil lowers triglycerides and raises LDL cholesterol [67]. However, additional studies are needed to gain a more comprehensive understanding of the role of fish oil supplementation in type 2 diabetes.

### 4.5. Inflammatory Disease

Due to their ability to regulate inflammatory processes and cellular responses, *n*-3 fatty acids have the potential to affect the development and progression of a multitude of diseases associated with an inflammatory state, including rheumatoid arthritis, Crohn’s disease, ulcerative colitis, type-1 diabetes, cystic fibrosis, asthma, allergic disease, chronic obstructive pulmonary disease, psoriasis, and multiple sclerosis (reviewed in [20,68,69]). In a rat model of experimental colitis [70], administration of an *n*-3 PUFA-enriched parenteral LE decreased colonic concentrations of pro-inflammatory mediators and attenuated the morphological and inflammatory consequences of colitis. Although the current state of knowledge is insufficient to support a clear recommendation for the use of *n*-3 PUFA in patients with inflammatory bowel disease, emerging evidence suggests a potential benefit. Clinical decisions regarding the use of fish oil in such patients should be informed by due consideration of the available evidence.

### 4.6. Immune Function

Among other effects, *n*-3 fatty acids inhibit immune and inflammatory functions by decreasing lymphocyte proliferation, cytokine production, natural killer (NK) cell cytotoxicity, and antibody production [71]. Additionally, *n*-3 fatty acids suppress neutrophil chemotactic responsiveness to leukotriene B4 [71,72], reduce antigen-presenting capability, and decrease expression of major histocompatibility complex II (MHC II) molecules of mononuclear phagocytes [73]. DHA has been shown to partly restore the oxygen-dependent bactericidal mechanisms of monocytes [74], and neutrophils treated with EPA and DHA showed enhanced antiparasitic activity against Plasmodium falciparum [75].

Reports on the effects of different *n*-3 fatty acids on cytokine secretion and immune cell activity are somewhat inconsistent. In mice fed a diet rich in EPA, plasma concentrations of TNF-α were increased [76], whereas others have demonstrated decreased TNF-α secretion by neutrophils in DHA-fed human volunteers [77]. Purasiri *et al.* [78] observed decreased production of interleukin-1 (IL)-1, IL-2, TNF-α, and interferon-γ by human neutrophils treated with *n*-3 fatty acids, while Chavali *et al.* [76] reported decreased expression of IL-6 and IL-10 by endothelial cells and monocytes in mice fed an EPA-enriched diet [76]. Diets rich in *n*-3 fatty acids have been shown to inhibit lymphocyte activation and modulate antibody production by B-lymphocytes [71]. Conversely, the proliferative response to T-cell mitogens was increased by *n*-3 fatty acids in an animal model of autoimmune disease [79]. In a rat model of antibody-mediated autoimmune disease, diets low in fat, deficient in essential fatty acids, or rich in fish omega-3 fatty acids were associated with a better prognosis and higher survival [80]. In general, *n*-3 PUFA inhibit NK cell and lymphokine-activated killer cell activities. DHA feeding inhibited NK cell activity in healthy men [77]; however, a diet rich in *n*-3 fatty acids augmented NK cell cytotoxicity in healthy rats [81].

*N*-3 and *n*-6 PUFA differentially influence the plasma free fatty acid profile, with consequent effects on neutrophil functions [82]. In an open-label randomized study of septic patients with markedly reduced neutrophil function, patients receiving *n*-6 lipid infusions experienced persistent or worsening abnormalities in plasma free fatty acids and impaired neutrophil function, while patients receiving *n*-3 lipid infusions showed a rapid switch in the plasma free fatty acid fraction to a predominance of EPA and DHA over AA, with rapid incorporation of *n*-3 fatty acids into mononuclear leukocyte membranes and subsequent suppression of pro-inflammatory cytokine generation. Additionally, neutrophil function was significantly improved following administration of *n*-3 LE [82].

Collectively, these findings suggest that *n*-3 PUFA have a favorable effect on immunocompetence and inflammation and may therefore reduce the risk of clinical sequelae in critically ill septic patients.

## 5. N-3 Fatty Acids in Parenteral Nutrition—Clinical Benefits

### 5.1. Preservation of Hepatocellular Integrity During Long-Term PN in Adults and Children

Patients with critical illness, compromised gastrointestinal tract, sepsis, or recurrent infection who require prolonged PN therapy are at high risk of developing hepatic complications [83,84,85]. The long-term use of IVLE, especially at doses exceeding 1g/kg of body weight per day, is thought to play a role in the etiology of intestinal failure-associated liver disease (IFALD) [7,86]. Emerging evidence suggests that reducing the amount of *n*-6 fatty acids from soybean oil by partial replacement with *n*-3 PUFA from fish oil may improve parameters of liver function in parenterally fed adult and pediatric patients [87,88,89,90,91,92,93]. Thus, replacing a pure soybean oil emulsion by an LE containing soybean oil, MCT, olive oil, and fish oil (resulting in a lower *n*-6/*n*-3 fatty acid ratio of ~2.5:1) appears to be a promising approach [7]. Indeed, the available evidence supports the use of *n*-3 PUFA in PN, infused either as a part of the basic LE or added separately (as a fish oil emulsion supplement). Studies evaluating both short- and long-term use of *n*-3 PUFA-containing LE have demonstrated preserved hepatic integrity and improvements in parameters of liver function in adult and pediatric patients [7,89,90,94]. Klek *et al.* investigated the safety and tolerability of a soybean/MCT/olive oil/fish oil emulsion in patients with intestinal failure receiving long-term PN. After four weeks of treatment, measures of liver function (alanine transaminase [ALT], aspartate transaminase [AST], and total bilirubin) were significantly improved in patients who received the soybean/MCT/olive oil/fish oil emulsion compared with controls who received a conventional soybean oil emulsion [7]. Parenteral infusion of *n*-3 PUFA from fish oil has also been repeatedly shown to be effective in reversing PN-associated cholestasis in children when administered alone or in combination with a soybean oil-based lipid emulsion [95,96,97,98,99].

### 5.2. Critical Illness

According to the European Society for Clinical Nutrition and Metabolism (ESPEN) definition, a critically ill patient is a patient developing an intensive inflammatory response with failure of at least one organ (Sequential Organ Failure Assessment (SOFA) score >4) necessitating support of organ function during an ICU episode expected to be longer than three days [100]. Patients admitted to the intensive care unit (ICU) only for monitoring (typical ICU stay <3 days) are therefore not representative ICU patients. The aforementioned acute inflammatory conditions, SIRS, sepsis, and septic shock represent the range of abnormalities during critical illness.

Numerous studies in ICU patients confirm the clinical value of *n*-3 PUFA in critically ill patients. In a multicenter study in 661 critically ill patients with a Simplified Acute Physiology II Score (SAPS II) >32, Heller *et al.* demonstrated that IV fish oil improved survival compared with that predicted by the SAPS II score and reduced infection rates, antibiotic requirements, and length of stay in a dose-dependent manner [101]. Consistent with these findings, Mayer *et al.* concluded, based on a review of the available evidence, that inclusion of *n*-3 fatty acids in PN improves immunologic parameters and length of stay in surgical patients [102]. Finally, in a recent comprehensive meta-analysis of studies evaluating the use of *n*-3 PUFA in ICU patients, Manzanares *et al.* analyzed data from 10 RCTs involving 733 patients and showed that the use of fish oil-containing LE was associated with significantly fewer infectious complications (RR 0.64; 95% CI, 0.44–0.92; *p* = 0.02) [103]. Trends toward a reduction in the number of days on mechanical ventilation (weighted mean difference [WMD] −1.14; 95% CI, −2.67 to 0.38; *p* = 0.14; heterogeneity I2 = 0%) and the length of hospital stay (WMD −3.71; 95% CI, −9.31 to 1.88; *p* = 0.19) were also reported. However, there was no significant effect on ICU length of stay (WMD −1.42; 95% CI, −4.53 to 1.69; *p* = 0.37) or mortality (RR 0.90; 95% CI, 0.67–1.20; *p* = 0.46; heterogeneity I2 = 0%). In a subgroup analysis of trials including patients who received IV *n*-3 PUFA in combination with enteral nutrition (EN) [104,105,106,107], a trend toward a reduction in mortality was observed (RR 0.69; 95% CI, 0.40–1.18; *p* = 0.18; heterogeneity I2 = 35%) [103].

According to the ESPEN Guidelines on Parenteral Nutrition in Intensive Care, the addition of EPA and DHA to lipid emulsions has demonstrable effects on cell membranes and inflammatory processes, and fish oil-enriched LEs likely decrease the length of hospital stay in critically ill patients (grade B) [100]. Canadian recommendations endorse the use of second generation LE when PN is indicated [108].

### 5.3. Oncology

Preclinical studies have demonstrated that *n*-3 PUFA can be effective in preventing the initiation and progression of cancer cells [45]. In the clinical setting, it was recently shown that fish oil-supplemented PN may help to reduce inflammation and stabilize energy expenditure in patients with pancreatic cancer [49]. In the same study, fish oil also improved patients’ quality of life (QOL) by reducing cachexia, increasing appetite, and increasing performance status. In a separate study, fish oil-containing LE combined with gemcitabine improved the effectiveness of anti-cancer therapy in patients with advanced pancreatic cancer by decreasing gemcitabine resistance and improving QOL [49]. However, referring to a systematic review of the published studies evaluating EPA in cancer patients by Dewey *et al.* [109], ESPEN concluded that there was no benefit associated with the oral administration of EPA in patients with consolidated cachexia [110].

In recent years several well-designed clinical studies assessing the effect of nutritional intervention with EPA/fish oil in conjunction with antineoplastic therapy have been published. Positive results have be shown with regard to increased energy/protein intake and gain/maintenance of body weight/lean body mass (LBM) [111,112,113], with evidence to suggest a potential effect on several domains of health-related quality of life (HRQOL) [113]. Ma *et al.* recently published a systematic review examining the effects of *n*-3 fatty acids in patients with unresectable pancreatic cancer and reported a significant increase in body weight and LBM as well as a significant decrease in resting energy expenditure and improved overall survival (130–259 days *vs.* 63–130 days) in patients who consumed an oral nutrition supplement enriched with *n*-3 fatty acids compared to those who consumed conventional nutrition [114]. The review concluded that *n*-3 fatty acids are safe and appear to exert a positive effect on clinical outcomes and survival in pancreatic cancer patients.

### 5.4. Surgery

Recent studies have shown that the use of LE containing an increased dosage of *n*-3 PUFA may improve outcomes in surgical patients [115,116,117,118,119,120]. Among the clinical benefits observed in these studies were improvements in liver function tests [115,116], improvements in measures of immunologic function and inflammatory response [117,118], decreased length of hospitalization [116,119,120], and a reduced risk of clinical complications [116]. Moreover, the benefits of *n*-3 PUFA were observed when administered preoperatively (three days) [118], postoperatively (seven days) [116,117,119], or during the entire perioperative period [121]. Based on these findings, *n*-3 PUFA-enriched LE administered as a part of PN appear to confer benefits to selected groups of surgical patients, including those with major trauma or undergoing extended digestive tract resections or liver transplantation [122]. A summary of recent studies evaluating various IVLE formulations in surgical patients is presented in Table 1.

Several recent meta-analyses evaluating the clinical benefit of *n*-3 PUFA-enriched PN in surgical patients showed significant reductions in infectious complications [123,124,125,126,127], with three of these meta-analyses reporting a significant reduction in the length of hospital stay in surgical patients who received *n*-3 fatty acid-containing LE compared with controls [125,126,127].

### 5.5. Safety and Tolerability during Long-Term Use

Data from available studies confirm the safety and tolerability of *n*-3 PUFA-containing LE during long-term use, regardless of indication [7,91]. In adult patients with stable intestinal failure requiring long-term PN, beneficial effects were observed on parameters of liver function and Vitamin E profile following the administration of a mixed-type LE containing 15% fish oil for four weeks [7]. Safety and tolerability were also demonstrated in 28 children (age, five months to 11 years) with short bowel syndrome, chronic intestinal pseudo-obstruction, or congenital disease of the intestinal mucosa who required home PN for at least four weeks [91]. Finally, a meta-analysis of 23 trials involving 1503 patients found no evidence of deleterious effects or symptoms of intolerance during long-term use of fish oil-containing LE during ICU stay or surgical hospitalization [126].

### 5.6. Recommended Dosage and Duration of Intervention

According to ESPEN Guidelines on Parenteral Nutrition in Intensive Care, lipids should be an integral part of the regimen to provide energy and essential fatty acids in all patients requiring PN [100]. IVLE (LCT, MCT, or mixed emulsions) can be administered safely at a rate of 0.7–1.5 g/kg over 12 to 24 h [100]. In the previously mentioned multicenter trial by Heller *et al.*, IV fish oil was safe and conferred significant clinical benefits when administered to critically ill adults in doses between 0.1 and 0.2 g/kg/day [101]. Therefore, it seems reasonable to recommend the administration of fish oil in doses higher than 0.1 g/kg/day. Studies in surgical patients showed that while short-term (<5 days) administration of *n*-3 PUFA influences immune parameters and liver function, postoperative administration also reduces complication rates and length of stay [118,119]. While it takes several days for *n*-3 PUFA to be incorporated in host tissues and influence the generation of inflammatory mediators, impairment of host defense mechanisms occurs immediately after surgery. Braga *et al.* [128] thus concluded based on a literature review that *n*-3 PUFA should be given prior to surgery to achieve an optimum effect. In premature infants, a mixed LE with 15% fish oil administered for seven to 14 days showed a potential beneficial effect on cholestasis, *n*-3 fatty acids, and Vitamin E status [90,92,129].

### 5.7. Cost-Effectiveness

The cost-effectiveness of omega-3 PUFA-enriched LE is an important consideration, as acquisition costs for fish oil-containing LE are comparatively higher than those for pure soybean or MCT/LCT-based emulsions [130]. Nonetheless, pharmacoeconomic analyses support the supplementation of LE with *n*-3 PUFA. Wu *et al.* demonstrated that fish oil-based LE improves clinical outcomes and decreases the overall costs of ICU stay compared with standard LE in Chinese ICU patients (mean savings, ¥10,000) [131]. Additionally, in the most comprehensive analysis of the cost-effectiveness of LE published to date, Pradelli *et al.* analyzed patient outcomes and hospital economic data from Italian, French, German, and UK hospitals using a discrete event simulation scheme and demonstrated that treatment costs were entirely offset by reductions in antibiotic use and the length of hospital stay [132]. Cost savings ranged from €3972 to €4897 per ICU patient and from €561 to €1762 per non-ICU patient [126].

## 6. Conclusions

Lipids are not only an important energy source, but they also modulate metabolic processes at local, regional, and distant sites. IVLE are undoubtedly an indispensable element of PN regimens. Indeed, there is a strong scientific rationale for *n*-3 PUFA in PN; they improve clinical outcomes in pediatric, surgical, cancer, and critically ill patients as well as patients requiring long-term PN. Finally, LE-containing fish oils have a proven safety and tolerability profile and represent a cost-effective component of PN regimens.

## Figures and Tables

**Table 1:** Latest studies/meta-analyses evaluating the benefits of *n*-3 PUFA (polyunsaturated fatty acids) in parenteral nutrition in surgical populations *.

Author	Year	Population	Intervention	Duration	Result
Jiang [117]	2010	Colectomy and rectotomy (*n* = 206)	LCT vs LCT+fish oil	7 days post-surgery	Significant reduction in LOS and SIRS
Wang [115]	2012	Gastrointestinal surgery (*n* = 64)	MCT/LCT *vs.* LCT+fish oil	5 days post-surgery	Amelioration of liver function and immune status
Han [116]	2012	Major surgery (*n* = 38)	MCT/LCT *vs.* LCT+fish oil	7 days post-surgery	Reduced postoperative liver dysfunction and infection rate
Zhu [119]	2012	Colectomy and rectotomy (*n* = 57)	LCT *vs.* fish oil	7 days post-surgery	Reduced LOS
Zhu [120]	2012	Liver transplantation (*n* = 98)	Oral diet *vs.* standard PN vs fish oil PN	7 days post-surgery	Reduced incidence of liver injury, decreased LOS and infectious complications
Berger [121]	2013	Cardiopulmonary bypass surgery (*n* = 28)	Fish oils vs saline	12 and 2 h before surgery and after surgery	Decreased biological and clinical signs of inflammation
De Miranda Torrinhas [118]	2013	Surgery for gastrointestinal cancer (*n* = 63)	MCT/LCT *vs.* fish oil	3 days post-surgery	Significant increase in IL-10 levels (day 3), decrease in IL-6 and IL-10 levels (day 6), less decline in leukocyte oxidative burst
Chen [123]	2010	Major abdominal surgery, meta-analysis (*n* = 892)	Fish oil *vs.* various control emulsions	Various	Decreased LOS in the hospital and ICU, reduced postoperative infection rate, improved liver function
Li [124]	2013	Major surgery, meta-analysis (*n* = 1487)	Fish oil	Various	Decreased infection rate, LOS, and liver dysfunction; no effect on mortality
Pradelli [125]	2012	Subgroup analysis in patients undergoing major abdominal surgery and not admitted to ICU (*n* = 740)	*n*-3 PUFA-enriched *vs.* standard lipid emulsions	Various	Significant reduction in the infection rate and LOS
Tian [126]	2013	Surgical patients, meta-analysis (*n* = 306)	Fish oil/LCT/ MCT *vs.* LCT/olive oil	Various	No significant difference, fish oil less toxic to liver when compared to LCT or olive oil

* Adapted from Klek *et al.* 2015 [122]. Abbreviations: ICU = intensive care unit; LCT = long-chain triglycerides; LOS = length of stay; MCT = medium-chain triglycerides; PN = parenteral nutrition; RCT = randomized clinical trial; SIRS = systemic inflammatory response syndrome.
